# Analyzing the impact of a state concussion law using an autoregressive integrated moving average intervention analysis

**DOI:** 10.1186/s12913-020-05742-0

**Published:** 2020-09-24

**Authors:** Lihong Huang, Lindsay Sullivan, Jingzhen Yang

**Affiliations:** 1 Department of Biostatistics, Zhongshan Hospital, Fudan University, Shanghai, 200032 China; 2 Center for Injury Research and Policy, The Abigail Wexner Research Institute at Nationwide Children’s Hospital, 700 Children’s Drive, RB3-WB5403, Columbus, OH 43205 USA; 3 Discipline of Children’s Studies, College of Arts, Social Sciences, & Celtic Studies, National University of Ireland, Galway, Galway, Ireland; 4 The Ohio State University, College of Medicine, Columbus, OH 43205 USA

**Keywords:** Concussion, Children, Intervention time series, ARIMA

## Abstract

**Background:**

Existing studies analyzing the impact of state concussion laws have found an increase in concussion-related medical encounters post-law, in some instances, such increases were observed during the pre-law period due to a potential “spillover” effect. This study assessed the effects of Ohio’s concussion law, while accounting for such a “spillover” effect, on the trends in monthly rates of concussion-related medical encounters in Medicaid insured children using autoregressive integrated moving average (ARIMA) analysis.

**Methods:**

We analyzed claim data obtained from the Partners For Kids database, a pediatric accountable care organization in Ohio. Concussion-related medical encounters for Medicaid-insured children (ages 0–18 years) treated between April 1, 2008 to December 31, 2016 were selected and analyzed. We assessed pre- and post-law trends in concussion-related medical encounters using an ARIMA intervention model. We also used traditional regression methods to validate the study results.

**Results:**

A total of 16,943 concussion-related medical encounters sustained by 15,545 unique patients were included. Monthly rates of concussion-related medical encounters significantly increased from 4.64 per 10,000 member months during the pre-law period to 6.69 per 10,000 member months in the post-law period (*P* < 0.0001). Three upward breaks in the monthly rates of concussion-related medical encounters were observed between 2009 and 2016, with two breaks observed during the pre-law period. Specifically, the increased breakpoint observed in July 2011 (*P* = 0.0186) was significantly associated with an estimated 7.3% increase (95% CI: 1.1–13.7) in the rate of concussion-related medical encounters. This finding was confirmed in the Poisson regression and curve fitting models. Furthermore, a seasonal trend in concussion-related medical encounters was observed with the highest rates in September and October of each year.

**Conclusions:**

Two of the three upward breaks identified in the monthly rate of concussion-related medical encounters occurred before the enactment of Ohio’s concussion law, suggesting a potential “spillover” effect. Further research is needed to confirm such an effect in children with other types of medical insurance.

## Background

Concussion, representing the immediate and transient symptoms of traumatic brain injury (TBI), is a form of mild TBI caused either by a direct blow to the head, face, neck or a blow elsewhere on the body with an impulsive force transmitted to the head [1–3]. Roughly 4000 concussions are sustained daily by US children aged ≤18 years [4]. Concussions often result in the rapid onset of short-lived impairment of neurological function that appears to resolve spontaneously and could have potential long-lasting effects on one’s physical, cognitive, emotional, and/or sleep health. Untreated or improperly managed concussion may contribute to functional impairments and other long-term, severe health consequences [4–6].

To address the growing concern about concussion among youth, in 2009, Washington State enacted the Zackery Lystedt Law, the first state-level legal intervention aimed to mitigate the potential negative health consequences of concussion [6, 7]. By 2014, all 50 US states and the District of Columbia enacted similar state-level concussion laws. Most concussion laws include the following three core tenets: (1) concussion education for coaches, parents, and/or athletes; (2) immediate removal of an athlete from play when a concussion is suspected; and (3) written clearance from a health professional before an athlete can return to play [8–10].

Several recent studies have evaluated the impact of these laws on rates of concussion and concussion-related medical encounters. These include studies that evaluated the pre- and post-law trends in concussion rates using data collected from a national representative sample of high school athletes [11] or that analyzed concussion-related emergency department (ED) visits based on electronic health records [12]. Results show significantly increased concussion rates and concussion-related medical encounters from pre-law to post-law and, in some instances, such increases were observed during the pre-law period [7, 12, 13]. Such increases are largely attributed to increased concussion recognition and reporting due to mandatory concussion education for coaches, parents, and/or athletes [6, 11–13]. Additionally, Gibson et al. examined differences in concussion-related medical encounters between states with and without a concussion law using national insurance claim data and found significantly higher rates of concussion-related medical encounters in states with a law as compared to states without a law (*P* < .01) [6]. Interestingly, Gibson et al. observed a 75% overall increase in rates of concussion-related medical encounters during the study period among states without a law. Possibly, these observed outcomes (rates of concussion and concussion-related medical encounters) in states without a concussion law were due to “spillover” effects of other states’ concussion laws. However, a paucity of research has accounted for this potential “spillover” effect when analyzing the impact of state concussion laws.

### Autoregressive integrated moving average

Unlike traditional regression analysis, autoregressive integrated moving average (ARIMA), a classic method of time series analysis either with or without intervention analysis, can be used to measure the effect of an external or exogenous intervention on a time series while accounting for a potential “spillover” effect [14]. While the ARIMA model has been widely used in other fields, such as economics [15, 16], agriculture [17], and tourism [18, 19], very few studies have used the ARIMA intervention model to assess the effects of concussion laws on rates of concussion or concussion-related medical encounters. Trojian et al. used ARIMA to evaluate the effects of Connecticut’s concussion law on ED visits for sports-related concussion by high school athletes and found that the monthly rate of concussions treated in the ED increased from 2.5 to 5.9 cases from pre-law to post-law [7]. Zemek et al. used ARIMA to investigate annual and seasonal trends of rates of physician office and ED visits for pediatric concussion in Ontario between 2003 and 2013 [20]; results revealed a steep increase in concussion visit rates from 2010 onward. However, these studies were limited by the use of ARIMA to test the seasonal variation in concussion rates rather than to directly assess the effects of the policy intervention.

### Current study

The current study applies the ARIMA intervention model to (1) quantify the rates of concussion-related medical encounters over time from 2008 to 2016 (pre- and post-enactment of Ohio’s concussion law) among Medicaid insured children aged ≤18 years in Ohio and (2) measure the effect of Ohio’s concussion law on trends in monthly rates of concussion-related medical encounters from pre-law to post-law while accounting for the “spillover” effects of other state concussion laws. In addition, this study used traditional regression methods to validate the study results.

## Methods

### Study data and population

Data used for this study were obtained from the Partners For Kids (PFK) pediatric accountable care organization database which includes the date and type of medical encounter(s), diagnosis, procedure(s), medication(s), treating physician(s), and facilities [21, 22]. PFK contracts with Medicaid-managed care plans in 34 counties in central and southeast Ohio, providing healthcare to approximately 330,000 children aged 0 to 21 years. For this study, healthcare claims for concussion-related medical encounters by actively enrolled PFK members aged 0 to 18 years between January 1, 2008 and December 31, 2016 were analyzed. Visits to multiple medical providers in the same day were treated as one concussion-related medical encounter.

Concussions were identified using the International Classification of Diseases, Ninth and Tenth Revisions, Clinical Modification (ICD-9-CM and ICD-10-CM) codes for concussion: 850.0, 850.1, 850.11, 850.12, 850.2, 850.3, 850.4, 850.5, 850.9, and those beginning with S06.0 [6, 23]. Only patients with one or more of the above concussion codes were included in the analyses. Concussions with a co-occurring severe TBI diagnosis code(s) (2.8%) were excluded.

To ensure accuracy of the initial concussion-related medical encounter, the study inclusion criteria were defined as follows: (1) an injury was sustained between April 1, 2008 and December 31, 2016, and (2) the patient was continuously enrolled in PFK for at least 30 days prior to the initial concussion-related medical encounter [22]. For patients with multiple concussion-related encounters, at least 90 days without a concussion-related encounter was required to denote unique injuries. A waiver of informed consent was approved by the Institutional Review Board of Nationwide Children’s Hospital.

### Outcomes of interest

The main outcome was the trend in monthly rate of concussion-related medical encounters per 10,000 member months, calculated as the number of initial concussion-related medical encounters in a month divided by the total number of PFK members in that same month, and multiplied by 10,000.

### Time series analysis

The Box-Jenkins ARIMA intervention time series analysis was used to quantify the impact of Ohio’s concussion law on trends in the monthly rates of concussion-related medical encounters. Seasonal ARIMA models were specified to account for the inherent dynamics in the series, and were expressed as (p, d, q) and (P, D, Q). The p, d, and q specified the order of the autoregressive, differencing, and moving average processes of the regular noise model, while P, D, and Q corresponded to the parameters for the seasonality component [24]. A log transformation was applied to the monthly rates series to ensure the normality and homogeneity of variance of the residuals. The concussion law was treated as the intervention and coded as a binary variable (0 = pre-law, before April 26, 2013; 1 = post-law, on or after April 26, 2013).

To analyze the time series of monthly rates of concussion-related medical encounters, we defined S = 12, corresponding to 12 observations per year. We assessed stationarity and used plots of autocorrelation function (ACF) and partial autocorrelation function (PACF) to identify the six parameters in the model. Akaike Information Criterion (AIC) and Bayesian Information Criterion (BIC) were used to compare models; the optimal model was based on the lowest AIC and BIC values. The mean absolute percentage error (MAPE) was calculated to assess forecast accuracy and to select an optimum model; the lower the MAPE value the better data fit.

The Ljung-Box Portmanteau (or Q) was used to examine the randomness of residuals of the estimated model. We assessed the effect of the intervention by interpreting the coefficient *β* for the indicator variable. The percent change in the post-law period was estimated as exp.(*β*)-1.

Additionally, we validated the results of the ARIMA model using both traditional Poisson regression and curve fitting models (Additional file 1). We first estimated the monthly rate per 10,000 member months and rate ratio of concussion-related medical encounters by including two independent variables (pre- or post-law group and the dummy variable of month identification) in the Poisson models, with a pre-defined reference month (either April 2008 or April 2013). We then employed the traditional curve fitting method to assess the trends of yearly rates of concussion-related medical encounters, using the coefficient of determination (R^2^) to determine goodness of fit. Finally, we compared the results of the ARIMA intervention time series analysis to the findings from the two traditional methods. All data analyses were conducted using SAS 9.4 and the TSA and forecast packages in R statistical software [24, 25], as appropriate. A *P* < 0.05 was considered statistically significant.

## Results

### Monthly rates of concussion-related medical encounters

During the study period, we identified a total of 16,943 unique concussions that were sustained by 15,545 unique patients. Of these, 63.1% occurred in males with an average age of 11.63 years (*SD* = 4.91). Table 1 presents the frequency of concussion-related medical encounters during the study period by month. The annual frequency of concussion-related medical encounters increased from 1139 to 2472 between 2009 and 2016. The overall rate of concussion-related medical encounters during the study period was 5.58 per 10,000 member months, with rates significantly higher during the post-law period (6.69 per 10,000 member months) than the pre-law period (4.64 per 10,000 member months) (*P* < 0.0001).

**Table 1:** Number of concussion-related medical encounters by month, 2008–2016

Month	Year								
	2008	2009	2010	2011	2012	2013	2014	2015	2016
**January**	–	70	87	111	141	167	141	183	199
**February**	–	68	70	93	147	130	143	175	177
**March**	–	74	107	112	158	152	157	232	158
**April**	80	111	105	109	177	179	194	205	217
**May**	81	116	106	136	177	186	204	227	209
**June**	75	73	84	122	149	126	140	129	121
**July**	51	86	108	116	126	142	152	133	112
**August**	94	115	137	195	232	241	270	276	227
**September**	109	152	201	254	317	309	414	407	377
**October**	111	113	182	199	243	356	387	312	339
**November**	69	88	98	123	125	173	168	165	176
**December**	56	73	64	111	125	150	188	146	160
**Total**	726	1139	1349	1681	2117	2311	2558	2590	2472

### Trend analysis using the ARIMA intervention model

Results of the ARIMA intervention model (0,1,1)(2,1,0)_12_ diagnostics showed that the models provided a good fit to the data. More than 90 models were tested under the auto.arima function in R forecast package; AIC values, BIC values and MAPE were calculated for the partial intervention ARIMA models that were close to the selected model. Model (0,1,1)(2,1,0)_12_ had the lowest AIC, BIC, and MAPE (− 243.22, − 229.03 and 6.38, respectively) (eTable 1). The correlation values of the fitted model were not outside the 95% Confidence Interval (CI) limits, and the residuals errors were considered white noise (eFig. 1), indicating that this model was appropriate. The Ljung-Box test found that the autocorrelation coefficients were not significantly different from zero (*Q* = 22.51, *P* = 0.43).

**Fig. 1:**
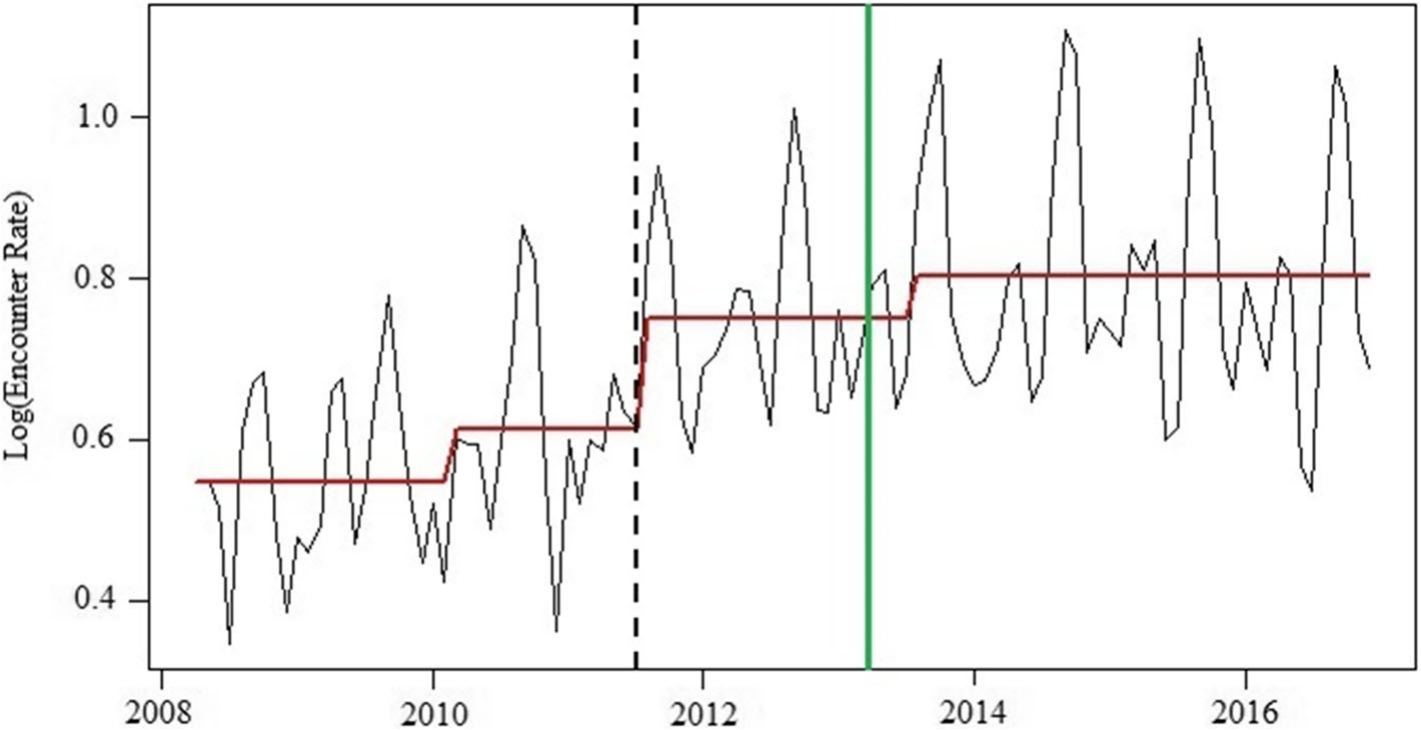
Three structural breaks in concussion-related medical encounter monthly rates series from 2008 to 2016. (The dashline is the significant increase in breakpoints, the solid green line is the month in which Ohio's concussion law was enacted, and the red line is the fitted levels by the three identified breakpoints)

**Fig. 2:**
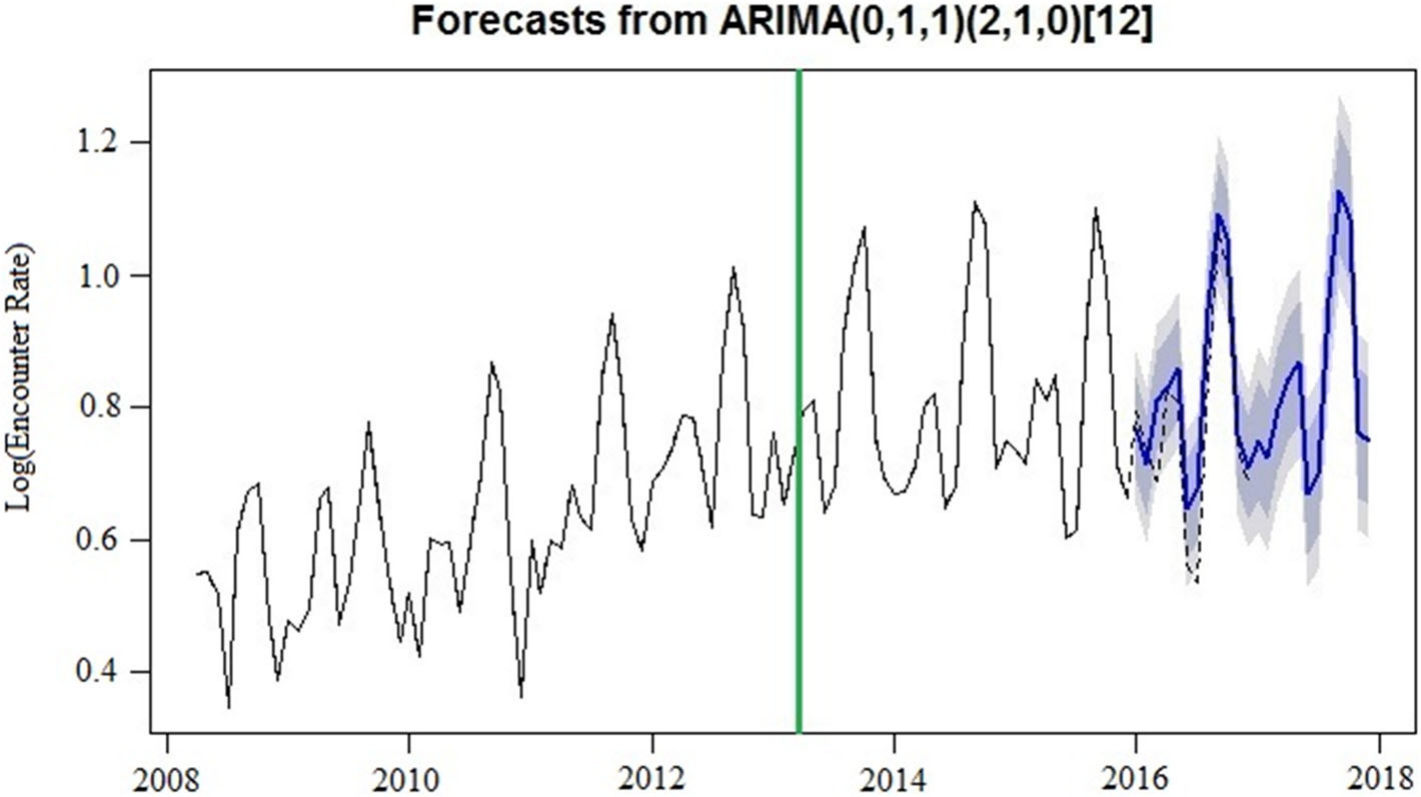
Forecasted 2016–2017 concussion-related medical encounters. (The solid blue line in 2016 and 2017 is the forecasted trend with 80 and 95% CI, the dashed line in 2016 is the observed encounter rate, and the solid green line is the month is which Ohio’s concussion law was enacted)

The monthly rates series had an upward trend between 2009 and 2015 (Figure 1). Three structural breaks were considered and tested: (1) February 2010, (2) July 2011, and (3) July 2013. As shown in Fig. 2, the expected rate series in 2016 and 2017, based on the ARIMA model forecast, were matched well with the observed trend, except for June 2016. Although the expected rates in June 2016 were lower than the observed rates, they were still within the 80% CI. The upward trends and forecast trends were not observed in 2016 or 2017. However, the seasonal trend remained, with September 2017 and October 2017 having the highest rates of concussion-related medical encounters.

### Potential “spillover” effects

The increased breakpoint observed in July 2011 (*P* = 0.0186) was significantly associated with an estimated 7.3% increase (95% CI: 1.1–13.7) in rate of concussion-related medical encounters, after controlling for the law intervention, trends, and seasonal variation (Table 2). The results from the curve fitting model demonstrated that the polynomial curve model was a good fit in estimating the trend of yearly rates of concussion-related medical encounters, with the fitted curve showing that the yearly rates increased from 2009 onwards (i.e., during the pre-law period) (eFigure 2). The Poisson model showed a significantly higher monthly rate of concussion-related medical encounters in September 2012 as compared to April 2013, with a rate ratio of 1.65 (95% CI = 1.38, 1.99) (eTable 2).

**Table 2:** Rate of concussion-related medical encounters per 10,000 member months from 2008 to 2016, autoregressive integrated moving average (ARIMA) intervention models with three breakpoints

Concussion Law Intervention Breakpoints		Change			ARIMA model and noise*		
		Percentage of Change	95% CI	*P*	Specification	Q24a	*P*
Period 1	Feb,2010	3.6	−4.0 to 11.8	0.3628	(0,1,1)(2,1,0)_12_	20.13	0.58
Period 2	July,2011	7.3	1.1 to 13.7	0.0186	(0,1,1)(2,1,0)_12_	21.70	0.48
Period 3	July,2013	2.0	− 4.4 to 8.8	0.5449	(0,1,1)(2,1,0)_12_	22.70	0.42

*The Ljung-Box Portmanteau (or Q-) test was used to test the randomness of residuals of the estimated models

## Discussion

To our knowledge, this study is the first to apply the ARIMA intervention time series analysis to evaluate the impact of Ohio’s concussion law on rates of concussion-related medical encounters over time among Medicaid-insured children aged ≤18 years. The ARIMA intervention time series model has several advantages over traditional time series analyses, including power, flexibility, and increased accuracy of predictions [26–29]. The findings suggest that the application of the ARIMA intervention time series analysis may be appropriate for explaining the effect of Ohio’s concussion law on concussion-related medical encounters. Our results revealed an increase in the monthly rates of concussion-related medical encounters from pre- to post-law, with two of the three upward breaks in the monthly rate of concussion-related medical encounters observed during the pre-law period. These results suggest that there was a potential “spillover” effect of other states’ concussion laws on concussion-related medical encounters in Ohio. Our results also showed a seasonal trend in the rate of concussion-related medical encounters, with rates highest in September and October of each year. Such findings may provide more precise and detailed information on the impact and nature of effect of Ohio’s concussion law on concussion-related medical encounters over time.

Although the traditional Poisson regression analysis could quantify rates ratios, comparing pre-law monthly concussion-related medical encounter rates to post-law rates, and the polynomial curve could describe the trend of yearly rates, these two traditional methods are limited by their ability to describe patterns of rate changes or forecast future trends of interest. ARIMA time series intervention analysis, on the other hand, has emerged as a standard statistical method to assess the impact of an intervention (i.e., a planned policy change) over time or in time series forecasting [19, 30, 31]. ARIMA time series intervention analysis has several advantages over traditional statistical methods (i.e., Poisson regression, a polynomial curve). These include that it is based on its own historical data [32] and the previous error terms for forecasting [33]; it allows for the identification and describing of temporal trends during the study period [34, 35]; and it enables the monitoring and forecasting of future monthly or annual trends [24, 33, 36]. However, it is important to note that the ARIMA intervention model is just one alternative method available to researchers when evaluating the impact of a law or forecasting trends of an outcome(s) of interest. The decision to employ the ARIMA time series model or traditional statistical methods should be guided by both the research question and the data type and structure.

The ARIMA time series intervention analysis has been increasingly used in epidemiologic research in recent years to assess intervention or policy impact [33, 34, 36]. Prior research shows that this model can be successfully applied to examine temporal trends and predict the incidence of various infectious diseases and injuries. For example, Lin et al. [33] used the ARIMA model to forecast monthly injury mortality trends and found that this model could be successfully applied to predict injury mortality. Despite its strengths and successful application in other fields [17, 19, 28, 29, 32], this study is the first to use the ARIMA time series intervention analysis to assess the impact of concussion laws on rates of concussion-related medical encounters over time from pre-law to post-law. Our findings may not only further our understanding of the effect of concussion laws on concussion-related healthcare utilization but may have important implications for future public health law impact research.

Using ARIMA intervention time series analysis, we identified three upward breaks in the monthly rates of concussion-related medical encounters during the study period. Two of these breaks were observed before Ohio’s concussion law went into effect. Such findings have not been previously reported; thus, further research is needed to confirm these findings. Possibly, the first identified break (February 2010) may have been influenced by the enactment of Washington State’s concussion law (The Zackery Lystedt Law) in 2009. Since the enactment of the Zackery Lystedt Law, media attention on and public awareness of concussion and the potential short- and long-term health consequences of concussion has increased dramatically throughout the US, which may partially explain the first observed increase in concussion-related medical encounters in Ohio. The second increase in concussion-related medical encounters was observed in July 2011. By then, 34 states had signed a state concussion law, 25 of which had been enacted [37]. These results may reflect a positive spillover effect of concussion laws; the law intervention in other states may have affected rates of concussion-related medical encounters in Ohio, perhaps highlighting the widespread effectiveness and benefits of the law intervention [38]. Finally, while not significant, we observed another increase in rates of concussion-related medical encounters in July 2013, immediately following the enactment of Ohio’s concussion law. As noted above, these three breaks may be the result of local and national policy efforts that aim to mitigate the potential negative consequences of concussion.

Consistent with previous research [7, 20], our results suggest a distinct seasonal trend in the rate of concussion-related medical encounters, with rates highest in September and October of each year. This finding was unsurprising given that American football is played during these months and prior research shows that American football has a high incidence of concussion as compared to other youth sports [39]. Although knowledge and awareness about concussion has increased in recent years in both athletes and non-athletes [11–13, 40], our findings, in line with others, suggest that additional preventive strategies (i.e., rule changes, reduction of player-to-player contact in practice sessions) aimed to mitigate the risk of concussion among youth athletes are needed [10, 11, 40]. These preventive strategies would be especially beneficial for youth athletes who play contact or collision sports such as American football, ice hockey, and soccer [11, 39–41]. Identification and testing of such preventive strategies may reduce the incidence of concussion.

This study has several limitations that warrant attention. First, similar to most studies that investigate the effects of policy changes on health outcomes, the present study is ecological in design. Because individual-level exposure data were not available, we were unable to attribute the increased rate of concussion-related medical visits solely to the concussion law. It is possible that the observed changes in rates of concussion-related medical encounter were due to unobserved economic or environmental confounding variables; thus, our results should be interpreted with caution. In addition, the observed upward breaks and seasonal trend in the monthly rates of concussion-related medical encounters were largely driven by our data and the selection of model(s) and need to be further validated. Finally, our results were based on medical encounters among Medicaid-insured children in Ohio; thus, our results may not be generalizable across states and/or to youth with other types of insurance.

## Conclusion

This study assessed the effect of Ohio’s concussion law on concussion-related medical encounters among Medicaid insured children and adolescents using the ARIMA intervention model. Results revealed an increase in monthly rates of concussion-related medical encounters during the study period, with two of the three upward breaks in the monthly rates of concussion-related medical encounters observed in the pre-law period. These findings suggest that there is a potential “spillover” effect of concussion laws in states without such laws. Our findings demonstrated that the ARIMA intervention model is a promising method to examine the effect of state concussion laws. Future studies should examine the effect of state concussion laws, including potential spillover effects and the effects of specific law element(s), on concussion-related medical encounters in children and adolescents with other types of health insurance.

## Supplementary information

**Additional file 1: Figure S1.** ACF and PACF of the transformed time series (A and B) and the residuals of the intervention ARIMA (0,1,1)(2,1,0)12 model (C and D). (DOCX 209 kb). Note: The correlation values of the fitted (0,1,1)(2,1,0)_12_ ARIMA intervention model were not outside the 95% Confidence Interval (CI) limits, and the residuals errors were considered white noise, indicating that this model was appropriate. **Figure S2**. Curve fitting for yearly rates of concussion-related medical encounters from 2008 to 2016. Note: A polynomial was fitted according to the trends in yearly rates. The coefficient of determination value (R^2^ = 0.9906) was close to 1, showing good goodness of fit. The fitted curve showed that the yearly rate of concussion-related medical encounters increased from 2009 to 2014, followed by a decrease in 2015 and 2016.

## Data Availability

The datasets used and/or analyzed during the current study are available from the corresponding author on reasonable request.

## Abbreviations

ARIMA: autoregressive integrated moving average
PFK: Partners For Kids
ACF: autocorrelation function
AIC: Akaike Information Criterion
BIC: Bayesian Information Criterion
MAPE: The mean absolute percentage error
PACF: Partial autocorrelation function
