# Supplementary material for: Analyzing the impact of a state concussion law using an autoregressive integrated moving average intervention analysis
Source: BMC Health Serv Res. 2020 Sep 24;20:898. doi: 10.1186/s12913-020-05742-0 (PMC7517644; doi:10.1186/s12913-020-05742-0)
Supplement: Supplementary file 1 — Additional file 1: Figure S1. ACF and PACF of the transformed time series (A and B) and the residuals of the intervention ARIMA (0,1,1)(2,1,0)12 model (C and D). (DOCX 209 kb). Note: The correlation values of the fitted (0,1,1)(2,1,0)12 ARIMA intervention model were not outside the 95% Confidence Interval (CI) limits, and the residuals errors were considered white noise, indicating that this model was appropriate. Figure S2. Curve fitting for yearly rates of concussion-related medical encounters from 2008 to 2016. Note: A polynomial was fitted according to the trends in yearly rates. The coefficient of determination value (R2 = 0.9906) was close to 1, showing good goodness of fit. The fitted curve showed that the yearly rate of concussion-related medical encounters increased from 2009 to 2014, followed by a decrease in 2015 and 2016. [file 12913_2020_5742_MOESM1_ESM.docx]

**Supplementary Material**

**Additional file 1.** Validating the results from the ARIMA intervention model using traditional Poisson regression and curve fitting models

We used the ARIMA model as the main statistical method in this study. We selected the best model using the auto.arima function in R forecast package. We calculated AIC and BIC values as well as the MAPE for the models that were closest to the selected (0,1,1)(2,1,0)_12_ model (i.e., the best model identified by the auto.arima function) (see eTable 1). The lowest AIC, BIC, and MAPE (-241.64, -229.03 and 6.38, respectively) was in Model (0,1,1)(2,1,0)_12._ eFigure 1 shows the autocorrelation function (ACF) and partial autocorrelation function (PACF) graphs for the time series and the (0,1,1)(2,1,0)_12_ ARIMA intervention model. The correlation values of the fitted model were not outside the 95% Confidence Interval (CI) limits (see eFigure 1C and 1D), and the residuals errors were considered white noise, indicating that this model was appropriate.

The results from the Poisson regression (see eTable2) showed a signiﬁcant increase in the monthly rates of concussion-related medical encounters from pre-law to post-law, with a rate ratio of 1.84 (95% CI 1.41, 2.39) when the first month of the study was used as the reference month (April 2008). The highest rate ratios were observed in September and October of each year from 2011 to 2016.

When we used the Ohio’s concussion law effective month (April 2013) as the reference month, we found a signiﬁcant decrease in the monthly rates of concussion-related medical encounters from pre-law to post-law, with a rate ratio of 0.79 (95% CI 0.64, 0.97). Although Ohio’s concussion law was not enacted until April 2013, the observed rate in September 2012 was significantly higher than the rate in April 2013, with a rate ratio of 1.65 (95% CI 1.38, 1.99). Following the enactment of Ohio’s concussion law, we observed increased rates in August, September, and October of 2013 and 2014 (eTable2).

For yearly rates of concussion-related medical encounters during the study period, the highest rate was observed in 2014. A polynomial was fitted according to the yearly rates trend:

$$y=-0.024x^{3}+0.303x^{2}-0.507x+3.746$$

The coefficient of determination value (R^2^=0.9906) was close to 1, showing good goodness of fit. The fitted curve showed that yearly rates of concussion-related medical encounters increased from 2009 to 2014, followed by a decrease in 2015 and 2016 (see eFigure2).

**eTable 1. AIC values, BIC values, and MAPE for different ARIMA intervention models**

| **Model** | **AIC** | **BIC** | **MAPE** |
| --- | --- | --- | --- |
| **(0,1,1),(1,1,1)_12_** | -233.87 | -226.31 | 6.70 |
| **(0,1,1),(2,1,0)_12_** | -243.22 | -229.03 | 6.38 |
| **(0,1,1),(3,1,0)_12_** | -241.22 | -228.62 | 6.41 |
| **(1,1,1),(2,1,0)_12_** | -241.32 | -228.71 | 6.38 |
| **(1,1,0),(2,1,0)_12_** | -224.35 | -214.26 | 7.03 |
| **(2,1,0),(0,1,1)_12_** | -228.87 | -218.79 | 7.73 |
| **(3,1,0),(0,1,1)_12_** | -233.82 | -221.21 | 6.51 |

(0,1,1),(2,1,0)_12_ had the lowest AIC, BIC and MAPE. The Ljung-Box test suggested that the autocorrelation coefficients of (0,1,1),(2,1,0)_12_ were not statistically different from zero (Q24a=22.51, *P*=0.43)

**eTable2: Poisson regression results for rates of concussion-related medical encounter from 2008 to 2016**

| **Variables** | **N** | **Membership** | **Rate Ratio**  **(95% CI)*** |  | **N** | **Membership** | **Rate Ratio**  **(95% CI)^Δ^** |
| --- | --- | --- | --- | --- | --- | --- | --- |
| **Legislation** |  |  |  |  |  |  |  |
| **Yes** | 9337 | 13959006 | 1.84 (1.41,2.39) |  | 9337 | 13959006 | 0.79 (0.64,0.97) |
| **Month** |  |  |  |  |  |  |  |
| **2010-09** | 201 | 273340 | 2.09 (1.61,2.70) | **2008-04** | 80 | 227011 | 0.57 (0.44,0.74) |
| **2010-10** | 182 | 274355 | 1.88 (1.45,2.45) | **2008-05** | 81 | 228175 | 0.57 (0.44,0.74) |
| **2011-08** | 195 | 281845 | 1.96 (1.51,2.55) | **2008-06** | 76 | 229380 | 0.53 (0.40,0.69) |
| **2011-09** | 254 | 291683 | 2.47 (1.92,3.18) | **2008-07** | 51 | 229916 | 0.36 (0.26,0.49) |
| **2011-10** | 199 | 288095 | 1.96 (1.51,2.54) | **2008-11** | 69 | 228793 | 0.49 (0.37,0.64) |
| **2012-04** | 177 | 289748 | 1.73 (1.33,2.26) | **2008-12** | 56 | 229998 | 0.39 (0.29,0.53) |
| **2012-05** | 177 | 291354 | 1.72 (1.32,2.24) | **2009-01** | 70 | 232595 | 0.48 (0.37,0.64) |
| **2012-08** | 232 | 307328 | 2.14 (1.66,2.76) | **2009-02** | 68 | 235975 | 0.46 (0.35,0.61) |
| **2012-09** | 317 | 308559 | 2.92 (2.28,3.73) | **2009-03** | 74 | 238263 | 0.50 (0.38,0.66) |
| **2012-10** | 243 | 298060 | 2.31 (1.80,2.98) | **2009-06** | 73 | 247191 | 0.48 (0.36,0.62) |
| **2013-04** | 179 | 288177 | 1.76 (1.35,2.29) | **2009-07** | 86 | 248634 | 0.56 (0.43,0.72) |
| **2013-09** | 309 | 300804 | 1.59 (1.32,1.90) | **2009-11** | 88 | 258458 | 0.55 (0.42,0.71) |
| **2013-10** | 356 | 301080 | 1.83 (1.53,2.18) | **2009-12** | 73 | 261528 | 0.45 (0.34,0.59) |
| **2014-09** | 414 | 321503 | 1.99 (1.67,2.36) | **2010-01** | 87 | 262991 | 0.53 (0.41,0.69) |
| **2014-10** | 387 | 324611 | 1.84 (1.55,2.19) | **2010-02** | 70 | 265324 | 0.42 (0.32,0.56) |
| **2015-06** | 129 | 323406 | 0.62 (0.49,0.77) | **2010-06** | 84 | 272194 | 0.50 (0.38,0.64) |
| **2015-07** | 133 | 322172 | 0.64 (0.51,0.80) | **2010-11** | 98 | 277278 | 0.57 (0.44,0.73) |
| **2015-09** | 407 | 323363 | 1.94 (1.63,2.31) | **2010-12** | 64 | 278397 | 0.37 (0.28,0.49) |
| **2015-10** | 312 | 321278 | 1.50 (1.25,1.80) | **2011-02** | 93 | 280780 | 0.53 (0.42,0.69) |
| **2016-06** | 121 | 327352 | 0.57 (0.45,0.72) | **2011-04** | 109 | 282347 | 0.62 (0.49,0.79) |
| **2016-07** | 112 | 325902 | 0.53 (0.42,0.67) | **2011-12** | 111 | 289737 | 0.62 (0.49,0.78) |
| **2016-09** | 377 | 325169 | 1.79 (1.50,2.13) | **2012-09** | 317 | 308559 | 1.65 (1.38,1.99) |
| **2016-10** | 339 | 325819 | 1.61 (1.34,1.92) | **2013-08** | 241 | 297780 | 1.65 (1.36,2.02) |
|  |  |  |  | **2013-09** | 309 | 300804 | 2.10 (1.74,2.54) |
|  |  |  |  | **2013-10** | 356 | 301080 | 2.42 (2.01,2.91) |
|  |  |  |  | **2014-08** | 270 | 319494 | 1.73 (1.42,2.10) |
|  |  |  |  | **2014-09** | 414 | 321503 | 2.63 (2.19,3.16) |
|  |  |  |  | **2014-10** | 387 | 324611 | 2.44 (2.03,2.93) |

Only estimations for “month” with P<0.0001 listed.

***Poisson regression model 1**: Reference for “month” was the first month of the study period (April 2008).

**^Δ^ Poisson regression model 2**: Reference for “month” was the first month after the enactment of Ohio’s youth concussion law (April 2013).


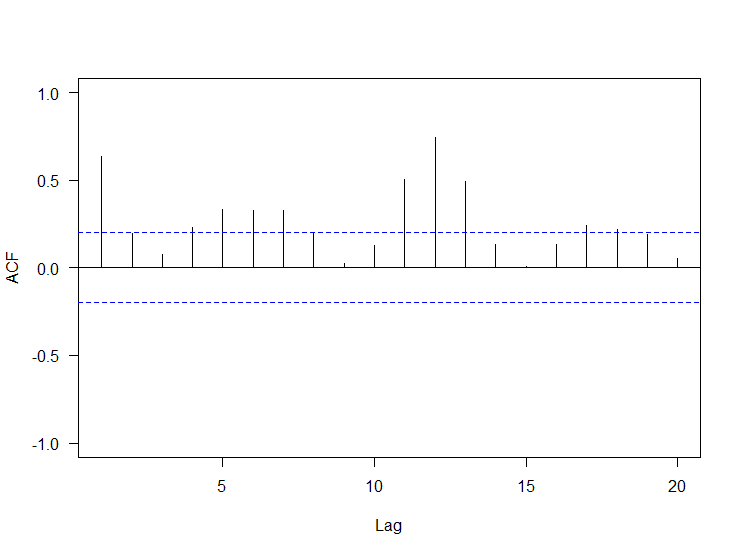

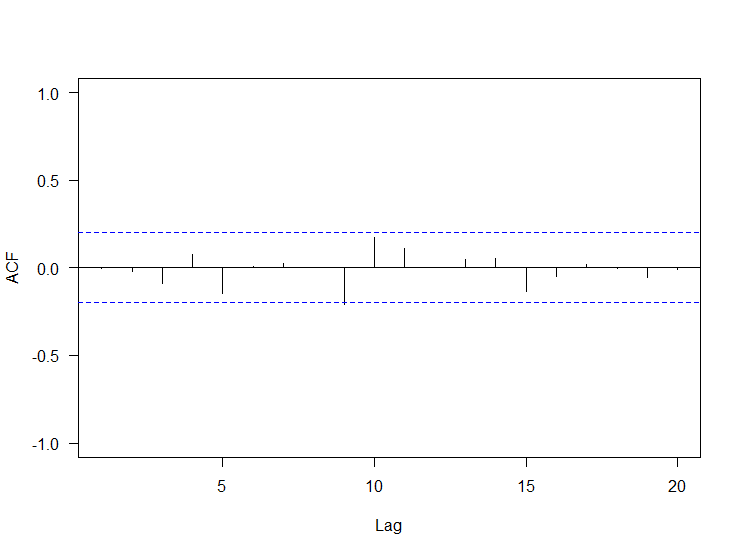

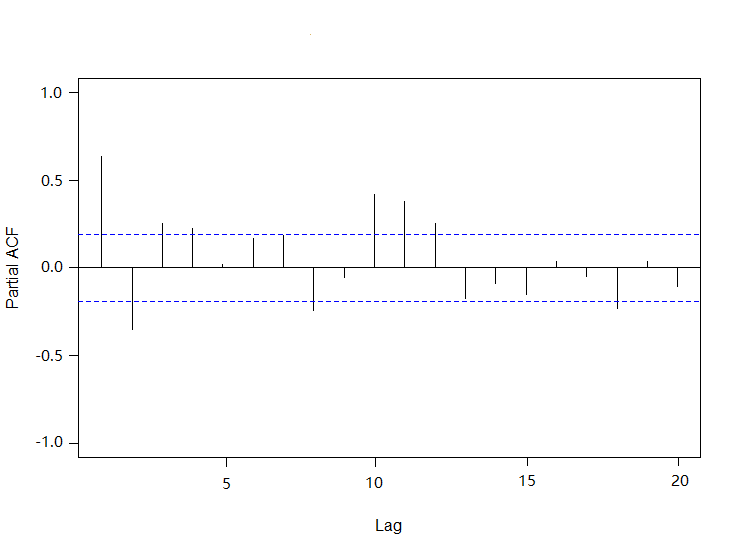

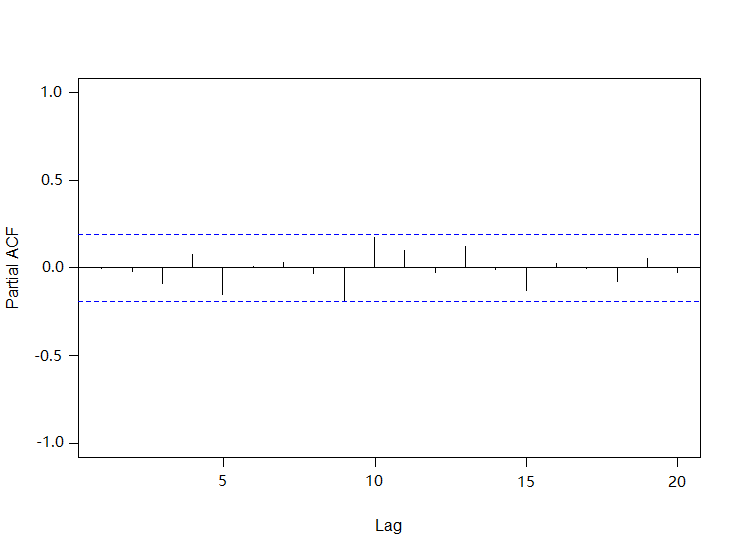


A

C

B

D

**eFigure 1. ACF and PACF of the transformed time series (A and B) and the residuals of the intervention ARIMA (0,1,1)(2,1,0)12 model (C and D).**

Note: The correlation values of the fitted (0,1,1)(2,1,0)_12_ ARIMA intervention model were not outside the 95% Confidence Interval (CI) limits, and the residuals errors were considered white noise, indicating that this model was appropriate.


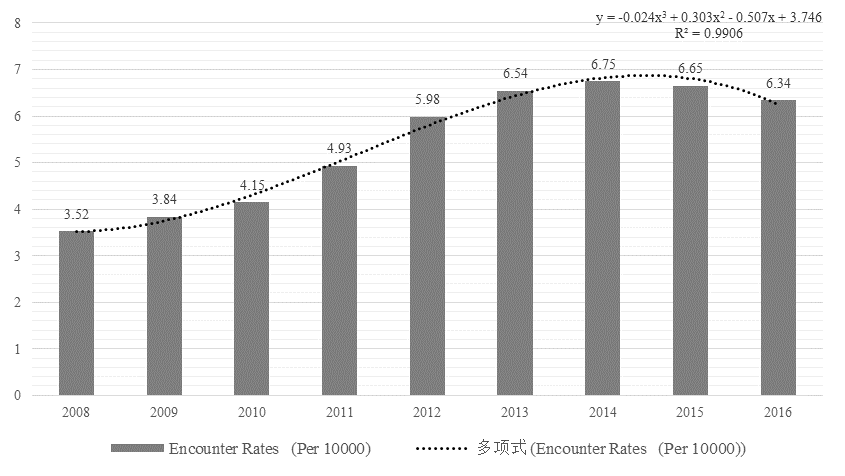


Polynomial Tendency

**eFigure2: Curve fitting for yearly rates of concussion-related medical encounters from 2008 to 2016**

Note: A polynomial was fitted according to the trends in yearly rates. The coefficient of determination value (R^2^=0.9906) was close to 1, showing good goodness of fit. The fitted curve showed that the yearly rate of concussion-related medical encounters increased from 2009 to 2014, followed by a decrease in 2015 and 2016.
